# Humility in medical practice: a qualitative study of peer-nominated excellent clinicians

**DOI:** 10.1186/s12909-022-03146-8

**Published:** 2022-02-09

**Authors:** Anupma Wadhwa, Sanjay Mahant

**Affiliations:** 1 Department of Pediatrics, University of Toronto and The Hospital for Sick Children, Toronto, Canada; 2 Child Health Evaluative Sciences, SickKids Research Institute, Toronto, Canada; 3 Institute of Health Policy, Management and Evaluation, University of Toronto, Toronto, Canada

**Keywords:** Humility, Doctor-patient relationship, Clinical expertise, Qualitative research

## Abstract

**Background:**

Humility has recently been conceptualized as a positive, multifaceted attribute in fields outside of medicine, such as psychology and philosophy. In medicine, there has been limited study into the nature of humility and its role in clinical practice. We sought to develop a deeper understanding of humility in medical practice through the lived-experiences of peer-nominated excellent clinicians.

**Methods:**

We conducted a qualitative study with secondary analysis of transcripts from individual open ended, semi-structured interviews of 13 peer nominated physicians [7 (54%) female] at an academic centre. Using constant comparative analysis, the transcripts were analyzed for instances where humility was discussed as it related to clinical practice.

**Results:**

Participants perceived humility to be an important driver for excellence in clinical practice. This was further explained using two overarching themes: an inward, intellectual perspective and an outward, social perspective. The physician’s inward perspective was their view of their abilities and limits, their self-confidence, and their intellectual openness and adaptability to the limitations and evolving nature of knowledge in medicine. Their outward perspective was an understanding and appreciation for the larger system in which they worked, an openness to others, and valuing patients’ experience. Through these perspectives, humility positively influenced clinical care, learning and curiosity, motivation in the care of others, and relationships with team members and patients.

**Conclusions:**

Humility in medicine is a rich, multifaceted construct that was perceived to be a driver for excellence in medical practice by peer-nominated excellent clinicians. Humility was seen as an active force in formulating and calibrating a clinician’s perspective of self and of others, and as such, positively influencing clinical practice. These findings will help inform a discourse in medical education and faculty development about the important role of humility in medical practice.

**Supplementary Information:**

The online version contains supplementary material available at 10.1186/s12909-022-03146-8.

## Introduction

Clinical expertise in medical practice is an important area of inquiry for medical education research and has direct implications for medical training and faculty development. Several areas of research have shed light on clinical expertise in medicine, such as the study of deliberate practice, adaptive expertise and clinical reasoning in medicine [1–3]. However, in order to contribute to a rich understanding of clinical expertise, further examination of excellent practice is required from varying perspectives.

In prior work, driven by an interest to understand clinical expertise and what makes an excellent clinician, we explored the personal and lived experiences of excellent clinicians [4]. We conducted a qualitative study using a grounded theory approach of peer-nominated excellent clinicians at one academic health science center. Through this work we developed a theory of the excellent clinician and found that humility was perceived as fundamental [4]. Similarly, Mylopoulos and colleagues in their study of renowned physician’s diagnostic practice found that humility was an important attribute in the reflective approach and practice-based learning [5]. Having a skeptical stance toward one’s own knowledge and viewing practice as a learning opportunity was important for a reflective approach in clinical practice and continuous learning [5].

Several studies have explored the importance and nature of physician humility in clinical practice through the perspective of patients. Huynh et al. found that physicians who were rated as generally humble in their interactions with their patients in primary care were rated as communicating more effectively with patients than physicians who were generally less humble [6]. Another study by Ruberton and colleagues found that patients’ rating of physician humility was associated with greater patient satisfaction, trust and self-reported health [7]. A cross-sectional study by Huynh et al. examined the nature of physician humility by asking participants to list humble behaviours that their doctor displayed [8]. Patient’s reported behaviours related to humility that fell into the themes of friendliness and approachability, non-verbal communication, patient focus, respect for the patient, and knowledge and admission of their own limitations.

Notably, despite these results from earlier studies and numerous commentaries emphasizing the importance of humility for clinical practice [9–11]., humility is not positioned prominently in existing medical training competency frameworks. This may in part be due to an ongoing lack of a deeper understanding of humility and its role in medical practice. Further study exploring humility in medical practice is needed.

Various types of humility have been described, many in fields outside medicine. For example, intellectual humility has been defined as “recognizing that one’s beliefs and opinions might be incorrect” or having the right stance towards one’s intellectual limitations [12, 13]. Cultural humility refers to a lifelong process of reflection and awareness of personal and cultural biases and a sensitivity to significant cultural issues of others [14]. Passionate humility has been described as the passionate conviction about being right, along with holding the possibility about being wrong [15]. Intentional humility refers to the intention of putting specific strategies in place to counter act the tendency to be self-centred and arrogant [16].

Of the emerging descriptions of humility, the framework described by Tangney stands out as it proposes a rich, in-depth description of this construct [17]. Through a review of theological, philosophical, and psychological literature, Tangney identified several key aspects to humility: an accurate assessment of one’s abilities; an ability to acknowledge one’s mistakes and limitations; an openness to alternate perspectives and contradictory views; keeping one’s accomplishments into perspective; a low focus on one’s self; and an appreciation and openness to learn the value of all things, including the different ways that people can contribute to our understanding [17].

The notion of humility, its dimensions and how it relates to performance in clinical medicine from the perspective of physicians has not been studied in-depth. Using sensitizing concepts of humility from fields outside of medicine, the objective of this study was to gain a deeper and more nuanced understanding of humility in medical practice by examining the lived-experiences of peer-nominated excellent clinicians. We discuss humility in medical practice through a secondary analysis of our qualitative study of excellent clinicians [4].

## Methods

### Study design

This is a secondary analysis of qualitative data originally collected from 2008 to 2010 in a grounded theory study designed to understand the nature of excellence clinicians, broadly, at an academic health science centre [4]. Then, in this secondary analysis conducted in 2019-2020, we further analyzed the transcripts of peer-nominated excellent clinicians to specifically gain a deeper understanding of humility in clinical practice. No new interviews were conducted for the present study. This secondary analysis of qualitative data [18] is an analytic expansion, “in which a researcher conducts a secondary interpretation of the qualitative data to answer new or extended questions”, according to Thorne’s classification of secondary qualitative research [19]. The first author is a pediatric infectious diseases specialist and the second author is a hospital general pediatrician. Both authors have training and experience in qualitative research and work in the practice setting of the study.

### Ethical considerations

Ethical approval for our study was obtained from the research ethics board at the Hospital for Sick Children, Toronto, Canada (REB Study No. 1000011884). In the primary study, written informed consent was obtained from study participants. Consent from the participants was not required for this secondary analysis, as no further interviews were conducted. All transcripts were de-identified.

### Study setting and participants

The study setting and participants have been reported [4]. The primary study was undertaken in a department of pediatrics at a pediatric academic health science center in Toronto, Canada, The Hospital for Sick Children. The Clinical Advisory Committee, Department of Pediatrics, nominated faculty in the department they perceived as being excellent clinicians. The Clinical Advisory Committee consists of 12 experienced physicians, chosen by the department chair, who represent diverse clinical environments and who are recognized for their expertise in clinical care. Members of the committee have an in-depth understanding of faculty clinical care performance through their clinical interactions and through the clinical performance reviews of faculty performed by the committee, which includes bedside clinical care and clinical scholarship. The Clinical Advisory Committee members were asked to list those faculty they felt were excellent clinicians. We did not specify specific criteria as we were trying to understand this construct in the broadest sense. There were 64 clinicians who received at least one nomination, 32 (50%) were female. Of the 64, 31 received one nomination, 12 received 2 nominations, 7 received three nominations, 4 received 4 nominations, 4 received 5 nominations, and 6 received 6 nominations. 20 of the 60 clinicians were from generalist type specialties (i.e. general pediatrics, emergency medicine, adolescent medicine, neonatal intensive care unit). We then sampled excellent clinicians for this study from those who had been nominated most frequently and to represent a range of specialties and both genders.

Interviews, that lasted at least 1 hour, were conducted by two research team members: 1 a physician faculty member in the Department of Pediatrics (SM) and the other a non-physician research team member who was not known to the faculty members. We deliberately formed a research team to include both a non-clinician who was not immersed in the practice setting as well as a clinician immersed in the setting in order to apply both an insider’s and outsider’s perspectives to the data collection and analysis. The varied experiences of the research team and thus the broadened perspectives served to maximize the potential interpretations of the data [20]. The interview script probed the participants on their notions of what attitudes, attributes, behaviors and skills constituted excellent clinical performance (see eSupplement 1). These concepts were probed through their experiences in their personal development and in their experiences with those whom they considered to be excellent clinicians. The interview script did not specifically ask about humility. However, as humility emerged as an important concept in interview after interview, we probed participants as they discussed it, in order to explore and elaborate its meaning and connection with clinical practice.

### Analysis

In the primary study, we conducted interviews and analyzed data concurrently, using a grounded theory approach, as our goal was to develop a theory of the nature of excellent clinicians. The research team met after the first, second and every 3rd interview to discuss coding and emerging concepts. The concurrent analysis and development of emerging theory informed concepts that were explored in subsequent interviews, consistent with a theoretical sampling approach that is used in grounded theory studies. We conducted interviews until we had a rich understanding of the concept of an excellent clinician and saturation of themes occurred at thirteen participants.

For this secondary analysis and in contrast with the analysis in the primary study, the meaning of humility from domains outside of medicine, such as psychology, and specifically as described by Tangney [17] served as sensitizing concepts [21]. For this secondary analysis, SM re-read all transcripts line-by-line and made memos to identify concepts and then codes relevant to understanding humility in clinical practice. In a similar approach to the analysis in the first study, we used a constant comparative approach, where-in concepts and codes were compared and contrasted within and between participants. The team members (SM and AW) discussed the codes, resolved differences by discussion, and then coalesced the codes into themes that provided an understanding of humility and its role in clinical practice.

### Trustworthiness

We employed several strategies in line with recommendations around increasing rigor and credibility in qualitative secondary data analysis [18]. First, our research team members were both involved with the primary study and knew the study context. There was a high degree of alignment between the focus of the first study, the data collected, and the goal of the secondary analysis in elaborating the meaning of humility in medical practice. Second, one of the research team members in this current study conducted the interviews, providing further insight into the data and interpretation. Third, the secondary analysis was conducted with uncoded transcripts. Fourth, memos and an audit trail of coding decisions were maintained and peer debriefing with the team members occurred throughout the analysis, to check confirmability.

## Results

Of the 13 clinicians interviewed, 7 (54%) were female, 2 (15%) were generalists, 7 (54%) were at the assistant or associate professor level, 4 (31%) were clinician investigators, 7 (54%) were clinician educators, 2 (15%) were clinician administrators, and 8 (62%) had been on faculty for more than 15 years. As of September 2020, all 13 (100%) participants had received at least one prestigious award for excellence in clinical care at the department, national and/or international level.

It emerged from the interviews that humility was seen as a multifaceted construct that was fundamental in clinical practice. As this participant stated, “It’s the inner morality of medicine” (P10: male, clinician educator, greater than 15 years of experience). Participants repeatedly and specifically identified humility and reflected on its central role in clinical medicine:

“I always say we’re as good as our last mistake. You can never rest in clinical medicine. Clinical medicine is a humbling experience, and if you don’t see it as such, you’re in danger.” (P10: male, clinician educator, greater than 15 years of experience).

They also spoke about other important attitudes, attributes and behaviours that they did not label as humility, but that we identified as meaning humility through our sensitized understanding.

Through our analysis, we explained humility and its role in clinical practice through two major themes, an inward, intellectual perspective and an outward, social perspective (Table 1). The inward, intellectual perspective referred to a view of one’s abilities and limits, one’s self-confidence, and one’s intellectual openness and adaptability to the limitations and evolving nature of knowledge in medicine. The outward, social perspective referred to an understanding and appreciation for the larger system in which they worked, an openness to others, and valuing patients’ experience. Through these perspectives, humility was perceived to positively influence clinical care, learning and curiosity, motivation in the care of others, and relationships with team members and patients. Humility was seen as an active, not passive force and required confidence, not meekness.

**Table 1:** Themes and Subthemes elaborating Humility

Theme	Description
**Inward or Intellectual perspective**	
One’s ability and limits	Insight to acknowledge one’s strengths and limitations in clinical care. Enables the clinician to learn through experiences and learn from mistakes.
Self-confidence	Confidence about what one knows, but also confidence to seek help when one doesn’t know.
Intellectual openness and adaptability to the evolving nature of knowledge in medicine	Appreciates not only personal limits of knowledge, but that knowledge in medicine is not finite, always changing, and has limits in what it can accomplish. Open to new and/or different perspectives, ways of thinking, ideas, and solutions.
**Outward or Social perspective**	
Appreciation for the larger system	Recognizes that one is just a piece of the larger system. While one’s role is important, one cannot do all nor have control over all and needs others.
Openness to others	Open stance to the views of patients and other colleagues. Focus on the well-being of patients as a central source of motivation. Treats and communicates with others with respect and empathy.
Valuing the patient’s experience	Respect for the patient’s experience and expertise in illness as a source of knowledge and learning.

In order to elaborate on the meaning and role of humility in clinical practice through the themes and subthemes developed from our analysis, we provide supportive illustrative quotes from the participants. Many of the themes and concepts were interconnected and interdependent.

### Inward, intellectual perspective

#### One’s ability and limits

The clinicians repeatedly spoke about how “part of being a good clinician” is the importance of “having insight into what your strengths and weaknesses are in the practice of medicine” (P5: female, clinician administrator, greater than 15 years of experience). As this clinician said:

“You have to know what you don’t know.” (P1: female, clinician educator, greater than 15 years of experience)

The insight to recognize and acknowledge one’s limitations and gaps in knowledge was discussed by many of the clinicians. This clinician explained how acknowledging and reflecting on her mistakes led to long-lasting learning:

“And the honest thing is, the thing you take home with you and learn from is the mistakes … you remember the things that … you didn’t do properly and that really changed something for the worse … you remember forever. And if you don’t do that I think that’s a problem” (P1: female, clinician educator, greater than 15 years of experience)

Having insight into one’s limitations was also linked with a sense of curiosity:

“ … it’s not necessarily just about knowing everything, but about how to [use] those gaps in your knowledge so that you seek out [the answers] … it’s related to curiosity, or drive.” (P13: female, clinician investigator, less than 15 years of experience)

As this clinician described, insight into gaps in knowledge in clinical practice were recognized, but rather than contributing to a negative self-perception, were used as a source of curiosity that motivated her to learn and seek solutions, leading to further clinical expertise development.

#### Self-confidence

Several clinicians also emphasized that humility did not mean a lowly feeling of one’s ability. It requires a “fine balance” between acknowledging one’s limitations and having confidence in one’s abilities in order to develop clinical expertise. Consider the following statement:

“Well, I think anyone who has been in clinical medicine for a while … will know that they need to be humble because if they’re not, if they’re arrogant or over-confident then they will make a mistake.” (P10: male, clinician educator, greater than 15 years of experience).

Interestingly, this participant then went on to qualify this statement with the following:

“We need to be careful a little bit, you obviously have to have a certain amount of confidence … otherwise you’re totally ineffectual … ” (P10: male, clinician educator, greater than 15 years of experience).

Another participant described how confidence was required in order to acknowledge personal limitations and seek help:

“I think you’ve got to be able to have enough confidence to say listen, I do not know that and I have to look it up, I have to go to somebody else. Be willing to ask other people, be willing to write around, to ask.” (P1: female, clinician educator, greater than 15 years of experience)

#### Intellectual openness and adaptability to the evolving nature of knowledge in medicine

In addition to appreciating one’s personal limits of knowledge, humility was also linked to having an intellectual awareness of the limitations of knowledge in medicine and its ongoing evolving nature.

This clinician reflected on the current state of knowledge in medicine and the limits to what it can accomplish in certain circumstances:

“ we don’t [always] cure disease, we convert acute disease into either chronic disease or maybe a more chronic compromised state.” (P3: male, clinician educator, less than 15 years of experience)

Further, this stance of understanding that knowledge in medicine is not static or absolute was seen as fundamental to lifelong learning and improving as a clinician.

“As you get older … you realize the stuff that you thought you knew, you didn’t really know, or what you learned 20 years ago has all been changed now as new information comes out, new approaches, molecular diagnoses, all those things. The more you know, the more you realize how little you actually know in the scheme of things.” (P11: female, clinician educator, greater than 15 years of experience)

This clinician further explained:

“The person who thinks that they can sit back and rest on their laurels, that they’ve done everything that they could possibly do and they’re as good as they can ever be: … it’s time for you to retire, or find a new career. There is always something we can learn and that’s what I try to teach the fellows, there isn’t a week that goes by in this institution that I don’t.” (P11: female, clinician educator, greater than 15 years of experience)

Acknowledging the limitations of knowledge in medicine and science was also seen as important for diagnostic expertise in clinical practice. Being open and adaptable to new knowledge, re-evaluating thinking, and considering different solutions was also emphasized, as described in the diagnostic reasoning process by this clinician:

“ … you have to be very humble about this whole thing, because certainly in the diagnostic work-up, if you pursue a certain pathway and you stick to it, you don’t remain open and flexible, that’s where you’re going to make mistakes.” (P9: female, clinician investigator, less than 15 years of experience)

Through their explanations of excellent clinical practice, such as those described above, the participants described the need for humility in the form of intellectual openness and adaptability towards the limitations and evolving nature of knowledge in medicine. This further contributed to motivation for lifelong learning and was seen as integral to effective clinical and diagnostic reasoning.

### Outward, social perspective

#### Appreciation for the larger system

In the interviews, the clinicians did not focus on their role in clinical care or accomplishments as singular. They emphasized the importance and expertise of the broader team of health care professionals in providing excellent patient care. For example, this clinician noted the importance of ‘acknowledging that there is a team … being aware that there are a group of people who all have valuable input’. (P4: male, clinician educator, less than 15 years of experience).

Similarly, this clinician spoke about the role of humility in recognizing the value of the collective expertise of the team:

“ … it gets back to I think the respect issue, or the humility issue. I think [excellent clinicians] all recognize that other people have contributions to make or are experts in areas that they might not be.” (P3: male, clinician educator, less than 15 years of experience)

#### Openness to others

Closely linked to the appreciation of the larger system and to recognizing one’s limits is having an openness to others. As this clinician explained:

“You can’t be good unless you recognize that you’re never going to have [all] the answers, that you might be wrong, that there isn’t one way of doing things, that there are other people that are going to have the answers, you’re not the one who always has the right answer all the time.” (P1: female, clinician educator, greater than 15 years of experience).

This openness to others was felt to extend to both healthcare colleagues, trainees and patients. For excellence in clinical practice, an open stance to others was recognized by many of these clinicians as critical to understanding people’s perspectives and the issues at stake to make good clinical decisions.

An open stance meant not only listening and genuinely considering the perspectives of other clinicians but breaking down hierarchies in medicine to improve open communication to understand a clinical problem or situation. Power hierarchies in medicine (e.g. senior doctor and junior doctor) might inhibit openness to others and communication, however, excellent clinicians are able to overcome these barriers, as this clinician described:

“There are hierarchies in medicine and I think some awareness of that is critical to the way that communication happens … But I think relying on those hierarchies to frame the communication is problematic. I think people … who break that down well, who allow people to overcome those barriers, allow more open discussion, listen properly to someone’s opinion, makes for a better clinician.” (P8: male, clinician educator, greater than 15 years experience)

Valuing the importance of understanding the context and narrative of the patient or colleague was felt to be an important way of developing a deeper understanding of any clinical situation and to therefore provide better care:

“So if you’re interested in the narrative, if you’re interested in, you know, what is bringing this patient here at this time and how did they get there … .you have a much better grasp of the situation.” (P8: male, clinician educator, greater than 15 years of experience)

For these excellent clinicians, a focus on others, specifically patients and their well-being, was an important source of motivation. While discussing from where her source of motivation and drive for clinical care originates, this clinician explained:

“It’s like really trying to get at, trying to make that difference by figuring out where you can help support the family.” (P5: female, clinician administrator, greater than 15 years of experience)

Furthermore, for excellence in clinical practice, the clinicians described how important respect and empathy were to develop effective and impactful relationships with patients, colleagues and trainees. This clinician emphasized the skill in being able to communicate at the same level of a colleague or patient to share knowledge and educate:

“I think they’re very good at not being condescending to people … People who are truly good clinicians, who are truly skilled don’t do that. They never make a family, or a patient, or a colleague feel that they are somehow beneath them or not worthy of their opinion or time. They’re able to educate, but not pontificate … they don’t act superior.” (P4: male, clinician educator, less than 15 years of experience)

#### Valuing the patient’s experience

For excellence in clinical practice, the clinicians spoke about the importance of being open to the perspective of patients not only to understand their health issues and concerns but also as a profound source of knowledge and learning for themselves as physicians. The patient was also teacher. This clinician described her genuine appreciation for the knowledge that patients bring and how they contribute to her learning:

“ … we don’t have all the science yet, now patients can teach us a lot too, and it comes back to an open mind, if we completely over assume we know everything, there is no way we will be flexible to realizing that we don’t know everything. I think it is quite amazing to see patients over years and they still teach you a lot … you learn how they’ve dealt with the disease even how the disease changes...” (P6: female, clinician investigator, less than 15 years of experience)

## Discussion

In this secondary analysis of a qualitative study of peer-nominated excellent clinicians in an academic center, clinicians saw humility as an important attribute linked to better performance in clinical practice. Humility was not a simple one-dimensional construct. Nor was it seen as traditional definitions have described – a modest view of oneself. Our analysis provides a deeper understanding of humility as a multifaceted construct and how it may be linked to excellence in clinical medicine. By formulating and calibrating one’s inward perspective of self and outward perspective of others, humility was perceived as a critical driver for these clinicians’ approach to clinical care, knowledge and learning, caring for others, and relations with healthcare professionals and patients.

For the clinicians we interviewed, humility was important for reasons beyond being perceived as ‘nice’; it was perceived to be important for developing clinical expertise and providing better patient care. Participants spoke about recognizing their limits and saw gaps in knowledge or skills as motivation and a source of curiosity to seek advice from others and learn. This is consistent with recent psychology research which has found that learners’ humility is associated with knowledge acquisition, including reflective thinking, curiosity, intellectual openness and in general is linked with an intrinsic motivation to learn [22]. The clinicians in our study also spoke extensively on the prosocial and affiliative feelings of appreciation for others. This was linked to a source of motivation and meaning for patient care. It was also connected with an openness to consider and value the perspectives and skills that others bring that is important for providing better patient care.

The meaning and concepts around humility in medical practice identified in our study map to many of Tangney’s key components of humility, which were developed from a review of the literature in psychology, philosophy, and theology [17]. The inward or intellectual perspective theme in our study relates to Tangney’s major concepts of having an accurate assessment of one’s abilities, acknowledging one’s imperfection, and keeping one’s accomplishments into perspective. The outward social theme in our study is aligned with the Tangney’s concepts around having an appreciation and openness to value of all things and the ways that other people can contribute to our understanding. Building on Tangney’s discussion of the meaning of humility, our study found that self-confidence is an important aspect to humility that is integral for effectiveness in clinical practice. Our findings also extend Tangney’s proposed construct of humility by providing how humility is perceived to be important in developing expertise in medical practice through the insights of peer-nominated excellent clinicians.

Our analysis does not establish what is core to the construct of humility. More recently, researchers within the field of positive psychology have conceptualized humility as a character strength that is important for humans to thrive. However, there is no clear consensus on what kind of construct humility is (e.g. a virtue, trait, or emotion) and what is at its core [23]. Wright et al. recently propose that at the core of humility is a “psychological positioning of oneself that is both epistemically and ethically aligned” [23]. Epistemically aligned refers to the perspective of oneself as a “finite and fallible being”, and so holding a limited ‘grasp’ or perspective on the ‘whole’ (low self-focus); ethically aligned refers to the perspective of oneself as just one among a many other ‘morally relevant beings’, whose perspectives are just as legitimate and worthy as one’s own (high other-focus). The dual dimensions of humility proposed by Wright et al. also align with the findings of our study in the context of medical practice. The theme of the inward, intellectual perspective speaks to the clinician’s realization that their knowledge and expertise in medical practice in incomplete (low self-focus) and the outward, social perspective speaks to their genuine need to help others and seek the collaboration and partnership of others (clinicians and patients) to provide excellent patient care (high other-focus).

Other researchers have challenged the notion that humility is all a positive construct and proposed that humility has two forms – a positive, appreciative form focused on others and also a negative, self-abasing form that is associated with shame, low self-esteems and submissiveness [24]. The participants in our study spoke to the idea that individuals must negotiate a fine balance to prevent being overtaken by feelings of self-limitation.

In our interviews, we did not explore how the academic medical centre’s culture around humility impacts clinical care excellence. Research from the management literature has found that organizations that embrace a culture of humility have more satisfied employees and better outcomes [25]. The patient safety movement emphasizes the importance of a safety culture, which includes aspects of humility such as understanding limits, learning from mistakes, and seeking help in the goal of improving care [26]. Understanding how the institutional culture inhibits or nurtures humility in medicine needs further study.

Our study has several limitations. First, we selected excellent clinicians through a peer-nominated process as other measures of individual excellence, such as quality of care measures, were not available to us for comparison across pediatric subspecialties. However, the fact that all the participants have received prestigious clinical excellence awards speaks to the recognition of their clinical excellence by other measures. Second, social desirability bias may have influenced the participants discussions around humility. Third, this was a single center study in one field of medicine. Studies in other contexts, such as community practice, primary care, and surgical specialties, are needed to understand the transferability of our results. Fourth, as this study was a secondary analysis of qualitative data, future studies on the importance of humility in clinical expertise conducted in these other contexts and using different data sources (e.g. field observations in addition to individual interviews) will be important to further elaborate and clarify the meaning of humility.

## Conclusions

Our findings from the experiences of peer-nominated excellent clinicians in one medical context suggest that humility is a rich, multifaceted construct that was perceived to be important for clinical practice in medicine. Humility formulates and calibrates a clinician’s perspective of self and others that then positively impacts their approach to medical practice. This is seen in their clinical care delivery, learning and curiosity, motivation in the care of others, and relationships with team members and patients. These findings add understanding to the different dimensions to humility in medicine and can inform a discourse in medical education and faculty development on humility and clinical expertise in medical practice.

## Supplementary Information

**Additional file 1.**

## Data Availability

The datasets used and/or analysed during the current study are available from the corresponding author on reasonable request.
