# Supplementary material for: Humility in medical practice: a qualitative study of peer-nominated excellent clinicians
Source: BMC Med Educ. 2022 Feb 9;22:88. doi: 10.1186/s12909-022-03146-8 (PMC8826652; doi:10.1186/s12909-022-03146-8)
Supplement: Supplementary file 1 — Additional file 1. [file 12909_2022_3146_MOESM1_ESM.docx]

**eSupplement 1. Semistructured Interview Guide for Peer-Nominated Excellent Clinicians**

This study is looking at what makes an excellent clinician in an academic hospital. We would like to better understand the behaviors, skills, attributes, and attitudes of an excellent clinician. I would like to hear your thoughts on this.

1. To begin with, can you tell me a bit about your job profile? *Prompt:* specialty, job activity profile? years in practice? How many months do you spend on clinical service now versus early on in your career, clinics/week? other areas of interest (education, research, admin), what are your 2 or 3 areas of focus?
2. Thinking of someone who you consider to be an excellent clinician – What stands out to you about this person? *Prompt*: skills, attitudes, behaviours, attributes?
3. As you may know, you were nominated by your colleagues as an excellent clinician. What do you think stands out to them about you? *Prompt:* Why do you think your colleagues see you as an excellent clinician? What qualities in you do you think they value?
4. Thinking about a diagnostic dilemma you have recently encountered. Can you tell me how you approached that? *Prompt:* Any pearls or advice for a junior colleague?
5. How about a challenging patient encounter you have recently experienced? Can you tell me how you approached that?
6. Do you enjoy your work as a clinician? What motivates you to excel at clinical care?
7. What techniques do you use or have you used to develop yourself as a clinician? *Prompt:* Tell me about some of the skills you have developed? Where did you learn these skills from? Can you tell me about any promotors or barriers in your development?
8. Suppose you are writing a handbook on being an excellent clinician in an academic hospital – what are some of your main topics you’d want to cover?
